# Risk perception and avoidance of preventive behavior on the COVID‐19 among cancer patients

**DOI:** 10.1002/hsr2.1401

**Published:** 2023-07-08

**Authors:** Mehdi Khezeli, Asghar Tavan, Sajjad Narimani, Vahideh Hoseini, Elham Zare Hosseinzadeh, Parisa Motamedi

**Affiliations:** ^1^ Social Development and Health Promotion Research Center, Health Institute Kermanshah University of Medical Sciences Kermanshah Iran; ^2^ Health in Disasters and Emergencies Research Center, Institute for Futures Studies in Health Kerman University of Medical Sciences Kerman Iran; ^3^ Department of Health in Disasters and Emergencies, School of Health Management and Information Sciences Iran University of Medical Sciences Tehran Iran; ^4^ Department of Nursing and Midwifery, School of Nursing, Social Determinant of Health Research Center Ardabil University of Medical Sciences Ardabil Iran; ^5^ Alavi Hospital Ardabil University of Medical Sciences Ardabil Iran; ^6^ Department of Infectious Diseases Faculty of Alborz University of Medical of Sciences Alborz Iran; ^7^ Department of Gynecology Ardabil University of Medical Science Ardabil Iran

**Keywords:** cancer patients, COVID‐19, defensive avoidance, preventive behaviors, risk perception

## Abstract

**Background and Aims:**

Willingness to engage in preventive behaviors against coronavirus disease 2019 (COVID‐19) depends on people's risk perception. This is especially important in cancer patients who are at risk of complications from the disease. Therefore, this study was conducted to investigate avoidance of COVID‐19 preventive behavior of in cancer patients.

**Methods:**

This cross‐sectional analytical study was done with 200 cancer patients who were selected by convenience sampling method. The study was conducted in Imam Khomeini Hospital of Ardabil, Iran from July to August 2020. A researcher‐made questionnaire was used to investigate the risk perception of cancer patients towards COVID‐19 with seven subscales according to the Extended Parallel Process Model. Data were analyzed by SPSS 20 using Pearson correlation and linear regression tests.

**Results:**

The mean and standard deviation of the age of 200 participants (including 109 men and 91 women) was 48 ± 17. Results showed that the response efficacy (12.6 ± 2.2) had the highest mean and defensive avoidance (8 ± 2.8) had the lowest mean score among EPPM constructs. Linear regression results showed that fear (*β* = 0.242, *p* > 0.001), and perceived severity (*β* = 0.191, *p* = 0.008) were significant predictors of defensive avoidance.

**Conclusion:**

Perceived severity and fear were significant predictors of defensive avoidance, and providing accurate and reliable news and information can be effective in reducing fear and promoting preventive behaviors.

## 1 INTRODUCTION

A novel coronavirus was first reported in Wuhan, China in December 2019, and following its rapid spread, the World Health Organization declared coronavirus disease 2019 (COVID‐19) global health emergency in January 2020.^1^, ^2^ From the beginning of the disease outbreak, Iran was one of the countries with high number of patients and deaths caused by COVID‐19 and faced many challenges in controlling the disease.^3^ Worldwide studies have shown that the mortality rate of COVID‐19 among hospitalized patients is 2%–5%,^4^, ^5^, ^6^ while in Iran, according to the Ministry of Health reports, the mortality rate of hospitalized patients is up to 15%.^7^, ^8^ The high mortality rate among hospitalized COVID‐19 patients is likely due to the presence of underlying chronic diseases in the general population.^9^

COVID‐19 is highly contagious and can be transmitted through aerosols, droplets and direct contact, and people with COVID‐19 may also have latent symptoms for up to 14 days.^10^ The highly contagious nature of disease threatens cancer patients,^11^ because most patients are immunosuppressed due to the disease and its treatment. Cancer patients have higher rates of severe illness, ICU admission, and mortality after COVID‐19 than the general population.^12^, ^13^ In Iranian culture, cancer patients usually expect their relatives to visit them. This reduces social distancing and can increase the risk of contracting a novel coronavirus.^14^ Attending the hospital for treatment and increasing the risk of covid‐19 is one of the most important problems for cancer patients.^15^

In the present study, it was somewhat differently from the previous studies and preventive behaviors were not investigated, but rather the effect of risk perception and efficacy appraisal on defensive avoidance in relation to protective behaviors of COVID‐19 was investigated. Risk perception is one of the determinants of disease prevention and treatment, and studies have shown that in the case of COVID‐19, risk perception has a significant relationship with compliance with preventive behaviors.^2^, ^16^, ^17^ One of the most widely used models in the field of risk perception is the extended parallel process model (EPPM),^18^, ^19^ which is commonly used in health communication campaigns when a message is trying to persuade an audience to adopt a healthy behavior.^20^ EPPM has seven constructs including perceived susceptibility, perceived severity, self‐efficacy, response efficacy, fear, defensive avoidance, and protection motivation. According to the EPPM, fear‐arousing messages may initiate two appraisals, (1) appraisal of the threat and (2) appraisal of the efficacy. The threat appraisal involves the severity and susceptibility to the risk. The efficacy appraisal also includes self‐efficacy and response efficacy. In situations where appraisal efficacy is low, risk perception is not in a favorable state and fear overcomes both, defense mechanisms (defensive avoidance) are activated against healthy behavior. In such a situation, the possibility of protective motivation and perform preventive behavior decreases.^21^

Considering the special problems of cancer patients, especially during the covid‐19 pandemic, and the importance of risk perception in performing/not performing preventive behaviors against covid‐19, the present study investigated the role of risk perception and efficacy appraisal on COVID‐19 defensive avoidance in contrast to preventive behaviors in cancer patients.

## 2 METHODS

### 2.1 The study setting and participants

This cross‐sectional study was conducted with 200 cancer patients admitted to the oncology ward of Imam Khomeini Hospital in Ardabil, Iran. Patients were included in the study based on convenience sampling method from July to August 2020. The sample conceived for this study was calculated using the results of study by Rezaei et al.^2^ According to the standard deviation of 3.04 in the mentioned study, considering *α* = 0.05, *d* = 0.5 and the effect size of 1.4, the sample size was determined as 200 people.

$$n={\left(\frac{{Ζ}_{1-\alpha /2\;}\times \delta }{d}\right)}^{2}.$$

The inclusion criteria were having cancer, and not being infected with COVID‐19 at the time of the study. Data were collected using a researcher‐made questionnaire on risk perception of cancer patients towards COVID‐19 based on an extended parallel process model (EPPM). The process of data collection was done after explaining the objectives of the study and obtaining oral informed consent from the patients.

### 2.2 Measurements and scoring

The questionnaire consisted of three parts: 16 questions about demographic variables; 18 questions related to COVID‐19 risk perception based on EPPM included six subscales: perceived susceptibility, perceived severity, self‐efficacy, response efficacy, fear and defensive avoidance, each of which was evaluated with three questions; and finally, three questions to measure behavioral intention. The questions of the EPPM constructs and behavioral intention were scored with a 5‐point Likert scale. The content validity of the questionnaire was checked using content validity ratio (CVR) and content validity index (CVI) through a panel of experts, including six health education experts, two epidemiologists, and one oncologist. In the CVR evaluation, experts commented on the necessity of the questions, and in the CVI, the simplicity, relevance, and clarity of the questions were evaluated. All questions of EPPM constructs and behavioral intention scale were approved by experts with CVI of 92% and 94%, and CVR of 94% and 88%, respectively. Internal consistency of the questions was approved with Cronbach's alpha coefficient of 0.75 and 0.90, for the EPPM scale and behavioral intention scale, respectively.

### 2.3 Statistical analysis

Data were analyzed using Statistical Package for the Social Sciences (SPSS‐20). Mean ± SD or rate (%) were used to present the descriptive statistics. To ensure the quality of the data, the normality of the data was checked. Also, outlier data were searched and managed through boxplot and scatterplot. Linear regression was also used to determine predictors of defensive avoidance behaviors in which DA was the dependent variable and other constructs of EPPM, intention, and demographic and contextual characteristics were independent variables. First, the univariate regression showed that the model constructs separately met the conditions to be included in the multiple regression model. Visual inspection of the observations showed that the data were independent. The normality of the data was confirmed by histogram and its results. Durbin–Watson (DW) statistics rejected the autocorrelation of data (DW = 2.140). Linear regression was used to identify the factors related to defensive avoidance, so that the demographic variables, constructs of EPPM, and intention entered into the stepwise model. In this study, a 95% (*p* < 0.05) significance level was adopted.

### 2.4 Ethical aspect of the study

This study obtained ethics approval from the Ethics Committee of Ardabil University of Medical Sciences (IR.ARUMS.REC.1399.164). Oral informed consent was obtained from all of the participants.

## 3 RESULTS

### 3.1 Descriptive results

In the present study, 200 cancer patients participated with mean ± SD of age of 48 ± 17. Half of the participants had an age range of 51–60 years old, 54.5% (*n* = 109) were men, 38% (*n* = 76) had nonacademic education, 27% (*n* = 54) lived in rural area and about one‐third of patients (32.5%, *n* = 65) had gastrointestinal cancer. More details on demographic and contextual variables are provided (Table 1).

**Table 1:** Demographic status of participants.

Variables			*N* (%)
Age	40–50		35 (17.5)
	51–60		100 (50)
	>60		65 (32.5)
Gender	Male		109 (54.5%)
	Female		91 (45.5)
Education	Academic		124 (62%)
	Nonacademic		76 (38%)
Residency	Rural		54 (27%)
	Town		35 (17.5%)
	City		111 (55.5%)
Involved organ	GI		65 (32.5%)
	Blood		40 (20%)
	Lung		37 (18.5%)
	Breast		32 (16%)
	Skin		11 (5.5%)
	Other		15 (7.5%)

The mean and standard deviation of the EPPM constructs were as follow: perceived susceptibility (11.4 ± 2.4), perceived severity (10.0 ± 2.4), self‐efficacy (12.3 ± 2.7), response efficacy (12.6 ± 2.2), fear (9.3 ± 3.5), and defensive avoidance (8 ± 2.8). Also mean and standard deviation of intention was (12.4 ± 2.2). According to these results, response efficacy had the highest mean and defensive avoidance had the lowest mean score. The questions related to each subscale and the obtained score are presented (Table 2).

**Table 2:** Mean and SD of Covid‐19 EPPM questions.

Questions	*N*	M	SD
**Perceived sensitivity** It is possible that I also get covid‐19 If I do not follow health protocols, I may get covid‐19 I have a high level of immunity and it is not possible for me to get coronavirus	200	11.4	2.4
**Perceived severity** Covid‐19 is a fetal disease If I get covid‐19, I may lose my life Coronavirus only kills people who do not follow health protocols.	200	10	2.4
**Self‐efficacy** I can easily use a face mask and gloves. I can stay home during the coronavirus outbreak and avoid unnecessary traveling. I can always wash my hands.	200	12.3	2.7
**Fear** Thinking about the coronavirus and its side effects scares me. Seeing and hearing the news about Coronavirus scares me. I feel panic and despair when I think of Coronavirus.	200	9.3	3.5
**Response efficacy** Wearing a face mask can prevent Covid‐19. Frequent hand washing and implementing social distancing can prevent Covid‐19. Staying home can help prevent Covid‐19.	200	12.6	2.2
**Defensive avoidance** I do not want to think about the dangers of the Coronavirus I do not want to do anything to prevent the coronavirus I think that home quarantine is unlikely to be required to prevent covid‐19.	200	8	2.8
**Intention** I am going to use gloves and a face mask. I am going to wash my hands regularly. I am going to stay at home as much as possible to reduce the risk of Coronavirus.	200	12.4	2.2

Abbreviations: COVID‐19, coronavirus disease 2019; EPPM, extended parallel process model; SD, standard deviation.

### 3.2 Regression results

In the regression model, *R* squared (*R* ^2^), and adjusted *R* squared (adj‐*R* ^2^) were 0.282 and 0.28, respectively, according to which, about 28% of the changes in the defensive avoidance behaviors explained by significant dependent variables (Table 3).

**Table 3:** Regression analysis of EPPM constructs for COVID‐19.

Model	Unstandardized coefficients		Standardized coefficients	*t*	Sig.
	B	Std. error	*β*		
Perceived severity	0.222	0.083	0.191	−2.669	0.008
Fear	0.192	0.056	0.242	3.414	0.001
Intention	−0.069	0.099	−0.055	−0.702	0.483
Response efficacy	−0.010	0.123	−0.008	−0.079	0.937
Perceived susceptibility	0.099	0.081	0.087	1.221	0.224
Self‐efficacy	−0.059	0.097	−0.057	−0.611	0.542

*Note*: Dependent variable: Defensive avoidance. *R* squared (*R* ^2^ = 282), and adjusted *R* squared (adj‐*R* ^2^ = 0.28).

Abbreviations: COVID‐19, coronavirus disease 2019; EPPM, extended parallel process model.

As shown in Table 3, among the EPPM constructs in addition to the behavioral intention variable, fear (*β* = 0.242, *p*‐value > 0.1), and perceived severity (*β* = 0.191, *p*‐value = 0.008), were significant predictors of defensive avoidance behaviors.

## 4 DISCUSSION

This study was conducted to investigate risk perception toward COVID‐19 and defensive avoidance against COVID‐19 preventive behavior in cancer patients. Main results showed that fear and perceived severity as two main components of risk perception were significant predictors of defensive avoidance.

Risk perception plays a central role in many models and theories of health behavior, including the Health Belief Model (HBM), Protection Motivation Theory, the Theory of Reasoned Action, and EPPM.^22^ In the present study, which was based on EPPM, risk perception was measured directly by the constructs of perceived sensitivity and severity and implicitly by the constructs of self‐efficacy, response efficacy, and fear. A study in Iran by Motayerzadeh et al.^23^ based on EPPM showed that perceived severity was a significant predictor of 5% for performing preventive behaviors against COVID‐19. Rezaei et al.^2^ also showed that for each unit of increase in risk perception (perceived susceptibility and severity), preventive behaviors against COVID‐19 increase by about 5%. A study in United States by Garfin et al.,^24^ also showed that greater perceptions of the risks from Coronavirus were associated with greater frequency of social‐distancing behaviors.

Some studies have investigated both variables of risk perception and fear. For example, the study by Yıldırım et al.^25^ in Turkey suggested that perceived risk and fear can significantly increase engagement in preventive behaviors during the novel coronavirus pandemic.

Fear in the present study was significant strongest predictor of defensive avoidance in cancer patients. Some studies have shown that people with underlying medical conditions report more fear of COVID‐19.^26^ Qian et al.^27^ in China also found that higher COVID‐19 risk perception was associated with increased fear and anxiety. Musche et al.^28^ in Germany also showed that diabetic patients had a greater risk perception and fear of COVID‐19 than the general population.

Some studies, however, have dealt with the issue from another aspect. They have shown that high risk perception is not always associated with positive results and sometimes leads to increased fear and adoption of defensive and avoidance behaviors. For example, a study showed that the risk perception of the epidemic disease is related to different perceptions, which can be referred to the reduction of fear or the exaggeration of the risk around COVID‐19.^29^ The results of a qualitative study by Alqahtani et al.^30^ in Saudi Arabia also showed that risk perception is the basis of the tendency to deny risks or react with exaggeration in terms of precautionary reactions related to COVID‐19. This is a vicious cycle because the higher risk perception of contracting COVID‐19 can lead to an increase in anxiety and fear of chronic patients and subsequently cause postponement of treatment sessions and increase in avoidance behaviors.^16^

In the present study, defensive avoidance was dependent variables influenced by perceived severity and fear. According to psychoanalytic theory, a defense mechanism is a psychological strategy that aims to reduce anxiety caused by potentially threatening impulses.^31^ Perceived stress and tension caused by preventive measures related to the COVID‐19 pandemic can stimulate psychological defense mechanisms.^32^ Defensive avoidance occurs when people block out feelings and thoughts related to a threat or ignore further information about it, for example, changing the TV channel or ignoring news related to COVID‐19.^33^

The results of this study can be used in designing health messages to reduce defensive avoidance and increase preventive behaviors against covid‐19 in cancer patients. By understanding that reducing fear and increasing perceived severity can reduce defensive avoidance, health messages can be designed and presented in a targeted manner.

This study was limited by the reliance on self‐report data. Participants in this research may have over‐reported or low‐reported their risk perception. However, the present study provided us with significant results regarding the effect of fear and perceived severity of Covid‐19 on defense avoidance mechanisms.

## 5 CONCLUSION

The defense mechanisms against covid‐19 prevention behaviors were significantly predicted by fear and the perceived severity of the disease. This demonstrates that fear and disease severity perceptions may not always correspond to preventive behavior. It is acclaimed that authentic and reliable news, information, and health education be delivered by the mass media and healthcare professionals to diminution fear and augment preventative behaviors of COVID‐19 in cancer patients

## AUTHOR CONTRIBUTIONS

**Mehdi Khezeli**: Conceptualization. **Asghar Tavan**: Methodology. **Sajjad Narimani**: Formal analysis; investigation; supervision; validation. **Vahideh Hoseini**: Writing—original draft. **Elham Zare Hosseinzadeh**: Writing—review & editing. **Parisa Motamedi**: Data curation; formal analysis.

## CONFLICT OF INTEREST STATEMENT

The authors declare no conflict of interest.

## TRANSPARENCY STATEMENT

The lead author Sajjad Narimani affirms that this manuscript is an honest, accurate, and transparent account of the study being reported; that no important aspects of the study have been omitted; and that any discrepancies from the study as planned (and, if relevant, registered) have been explained.

## Data Availability

All data generated or analyzed during this study are included in this article and can be requested from the corresponding author.
